# Stages of a Transtheoretical Model as Predictors of the Decline in Estimated Glomerular Filtration Rate: A Retrospective Cohort Study

**DOI:** 10.2188/jea.JE20200422

**Published:** 2022-07-05

**Authors:** Daisuke Takada, Susumu Kunisawa, Akira Kikuno, Tomoko Iritani, Yuichi Imanaka

**Affiliations:** 1 Department of Healthcare Economics and Quality Management, Graduate School of Medicine, Kyoto University, Kyoto, Japan; 2 Japan Health Insurance Association Kyoto, Kyoto, Japan

**Keywords:** transtheoretical model, chronic kidney disease, kidney injury, stage of change, more than 30% decline in estimated glomerular filtration rate

## Abstract

**Background:**

The transtheoretical model (TTM) is composed of the multiple stages according to patient’s consciousness and is believed to lead people to realize the importance of healthier behaviors. We examined the association of TTM stages with the decline of estimated glomerular filtration rate (eGFR).

**Methods:**

We used the annual health checkup data and health insurance claims data of the Japan Health Insurance Association in Kyoto Prefecture between April 2012 and March 2016. TTM stages of change obtained from questionnaires at the first health checkup and categorized into six groups. The primary outcome was defined as a more than 30% decline in eGFR from the first health checkup. We fitted multivariable Cox proportional-hazards model for time-to-event analyses adjusting for age, sex, eGFR, body mass index, blood pressure, blood sugar, dyslipidemia, uric acid, urinary protein, and existence of kidney diseases at first health checkup.

**Results:**

We analyzed 239,755 employees and the mean follow-up was 2.9 (standard deviation, 1.2) years. As compared with the stage 1 group, the risk of eGFR decline was significantly low in the stage 3 group (hazard ratio [HR] 0.77; 95% confidence interval [CI], 0.65–0.91); stage 4 group (HR 0.80; 95% CI, 0.65–0.98); and stage 5 group (HR 0.79; 95% CI, 0.66–0.95).

**Conclusion:**

Compared with the precontemplation stage (stage 1), the preparation, action and maintenance stages (stages 3, 4, and 5), were associated with a lower risk of eGFR decline.

## INTRODUCTION

Chronic kidney disease (CKD) has been a global health problem for many years, and its prevalence has reached approximately 10–15% worldwide among 500 million people.^1^ CKD progression is caused by many pathophysiological risks such as diabetes, hypertension, and systemic immune disorders.^1^ Recently, dietary and lifestyle modifications were found to affect renal function, so the Kidney Disease: Improving Global Outcomes (KDIGO) guideline now recommends that CKD patients should monitor and change their behaviors, including smoking cigarettes, their healthy weights, and daily physical activity.^2^ In fact, the evidence that being a past smoker compared with current smoker was associated with a decreased risk of CKD progression^3^ indicates that behavior change might slow the disease progression. However, an issue would be that changing such healthy behaviors does not seem easy in clinical settings. Recently, some integrative theories of psychotherapies have evolved to address this issue.

The transtheoretical model (TTM) of behavior change is one of the integrative theories that divide common people into five categories based on temporal dimensions.^4^ In general, people move through five stages from precontemplation to contemplation, and then preparation, followed by action and maintenance stages when they change their behavior. Recent studies have documented that TTM-based intervention improved adherence with lipid-lowering or antihypertensive drugs,^5^^,^^6^ and promoted healthy eating, exercise, and other healthy behaviors in a randomized controlled trial.^7^

Nevertheless, it is important to understand the precise mechanisms by which behaviors affect renal function. The first step would be to determine whether each stage is associated with CKD progression. Here, we examined whether CKD progression is associated with TTM stages using the health-checkup Japanese database.

## METHODS

### Database and target populations

We performed a retrospective analysis using annual health checkup data and health insurance claims data of employers in companies insured in the Japan Health Insurance Association in Kyoto prefecture, Japan. Annual health checkups of employees aged more than 35 years are mandatory until they lose their eligibility (eg, quittance/change jobs, moving to other area, or death).

### Inclusion and exclusion criteria of participants

We recruited employees who were between 35 and 75 years old and had two or more health checkups from April 2012 to March 2016. We excluded those who had any kidney disease or missing data in the first health checkup. Questionnaires were acquired in each health checkup and contained the information on prescribed drugs, healthy behaviors, and alcohol consumption. Kidney disease was defined by The International Classification of Diseases, 10^th^ Revision codes, such as N00-08, I70, and Q61, in the claims data.

### Baseline variables

TTM stages of a change obtained from questionnaires at the first health checkup were categorized into six groups by the question: “Do you intend to improve your lifestyle habits of diet and exercise?: do not intend to take action in the foreseeable future, regarded as stage 1; intend to change in the next 6 months, regarded as stage 2; intend to take action in the immediate future until the next month, regarded as stage 3; made specific overt modifications in their lifestyles within the past 6 months, regarded as stage 4; a prevent relapse, but they did not apply change processes as frequently as people in action, regarded as stage 5; no answer to the question (missing data), regarded as “no concern”. The lifestyle behavior changes, including smoking cessation, undertaking physical activity, and achieving a healthy weight at 1 year after the first health check-up, were obtained from questionnaires on the next health check-up: Those who are “quitting smoking” mean those who answered “Yes” in the previous year and answered “No” in the present year to the question, “Are you a heavy smoker? (A heavy smoker refers to those who have smoked a total of over 100 cigarettes or have smoked for a period of 6 months and have been smoking during the past month.)”. Those who are “Undertaking physical activity” mean those who answered “No” in the previous year and answered “Yes” in the present year to the question, “Are you in a habit of doing exercise to sweat lightly for over 30 minutes a time, two times weekly, for over a year?”. “Decrease in the amount of drinking” is determined according to the question, “How much do you drink per day?”. “Decrease in the frequency of drinking” means an answer to the question “How often do you drink? (sake, shochu, beer, wine, whisky, or brandy, etc)”. The contents of questionnaires were created by reference to “Standard medical checkup and health guidance programs” by the Japanese government: Ministry of Health, Labor, and Welfare.^8^

The covariates were classified into groups as follows: four groups based on age (35–45, 46–55, 56–65, and 66 or more years); four groups based on body mass index (BMI; thin: ≤18.5 kg/m^2^, normal: 18.5–25 kg/m^2^, pre-obesity: 25–30 kg/m^2^, and obesity: >30 kg/m^2^) according to the World Health Organization; five groups based on eGFR (≤15, 30–15, 45–30, 60–45, and >60 mL/min/1.73 m^2^); three groups based urinary protein (using dipsticks: positive (≥1+), trace (±), and negative), abdominal circumference (if male ≥85 cm, female ≥90 cm); five groups based on blood pressure (systolic blood pressure [SBP] ≥180 mm Hg or diastolic blood pressure [DBP] ≥110 mm Hg without drugs, SBP ≥160 mm Hg or DBP ≥100 mm Hg without drugs, SBP ≥140 mm Hg or DBP ≥90 mm Hg without drugs, normal without drugs, and with drugs); and four groups based on dyslipidemia (triglyceride ≥150 mg/dL or high-density lipoprotein cholesterol <40 mg/dL was defined to be abnormal with hypolipidemic drugs, abnormality without hypolipidemic drugs, normal with hypolipidemic drugs, normal without hypolipidemic drugs), diabetes (fasting blood sugar ≥110 mg/dL or hemoglobin A1c ≥5.6% was defined to be as abnormality with antidiabetic drugs, abnormality without antidiabetic drugs, normal with antidiabetic drugs, normal without antidiabetic drugs), and hyperuricemia (defined uric acid ≥8 mg/dL without drugs or with use of anti-hyperuricemias). The information for each medication use was extracted from questionnaires.

### Statistical analysis

The primary outcome for survival analysis was defined as a decrease of 30% or more in eGFR.^9^ The eGFR was calculated by the equation used by the Japanese Society of Nephrology.^10^ Patients were followed until the outcome or censored.

The Cox proportional-hazards model was used for time-to-event analyses to estimate the hazard ratios (HRs); 95% confidence interval (CI) were used for the primary outcome. Follow-up period data for patients were censored on the date of the last health checkup. The analysis used two types of models: model 1 *(without medication factors)*, adjusted for age, sex, BMI, abdominal circumference, eGFR, and urinary protein; and model 2 *(with medication factors)*, adjusted for age, sex, BMI, abdominal circumference, eGFR, urinary protein, blood pressure, blood sugar, dyslipidemia, and uric acid. All the covariates were detected at the first health checkup. Schoenfeld residuals were used to check the proportional hazards assumption. A two-sided significance level of 0.05 was used, and all analyses were conducted using R version 3.4.1 (R Foundation for Statistical Computing, Vienna, Austria).

Subgroup analyses were performed for the model 2 condition, where the analysis population was stratified by employees 1) whose eGFR categorized as >60, 60–45, or ≤45 mL/min/1.73 m^2^ and 2) who did not attend hospital because of diabetes (no medication to reduce blood sugar or insulin injection), and 3) who met 1 or more criteria for Japanese metabolic syndrome.^11^

Sensitivity analyses were also performed for the model 2 condition, where we excluded from the analysis population 1) employees aged 60 or more years or 2) employees who had taken any medication for hypertension, diabetes, or dyslipidemia. The former employees were excluded because retirement is more likely to occur 60–65 years in Japan, which may cause healthy workers bias, and we minimized the impact of employees lost to follow-up.

## RESULTS

**Figure 1.**
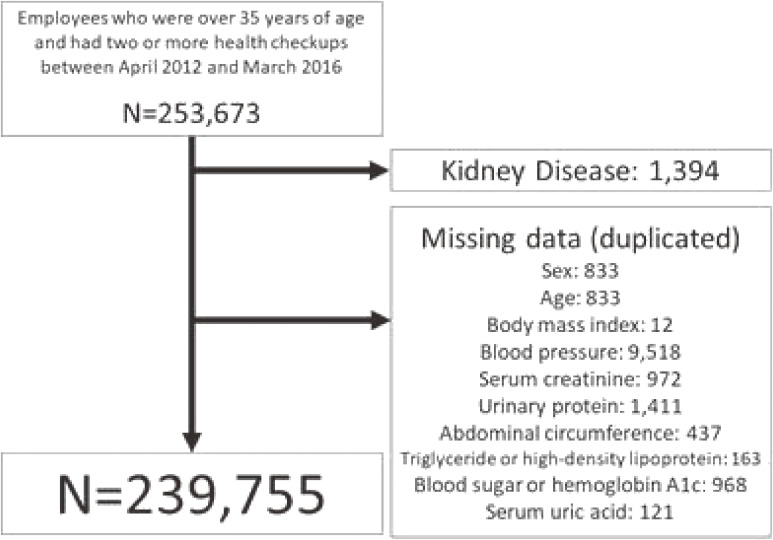
Flow chart for selection of study participants from the Japan Health Insurance Association in Kyoto Prefecture

A total of 253,673 employees were enrolled and fulfilled the inclusion criteria; 12,593 (4.9%) were excluded due to missing data and 1,392 due to the prevalence of kidney disease. We analyzed the remaining 239,755 employees (Figure 1). By the end of follow-up, there were 1,836 persons (0.8%) whose eGFR decreased 30% or more, and the mean follow-up was 2.9 (standard deviation, 1.2) years.

The characteristics of each stage are shown in Table 1. The group of stage 5 tended to have a higher serum creatinine and higher proportion of prescription drugs, including for diabetes and dyslipidemia. The proportion of physical activity changes at 1 year after the first health check-up tended to be higher in stages 3–5 than in stages 1–2. In particular, the proportion of undertaking physical activity was 8.0% in stage 3; 12.0% in stage 4; and 8.6% in stage 5, compared with 5.3% in stage 1; 5.2% in stage 2.

**Table 1.** Summary of patient characteristics for each stage of change according to the transtheoretical model

	Stage 1: Precontemplation (not ready)	Stage 2: Contemplation (getting ready)	Stage 3: Preparation (ready)	Stage 4: Action (current action)	Stage 5: Maintenance (monitoring)	Unknown: No concern
Total number	60,535	80,522	27,285	16,110	19,889	35,414
Sex, male (%)	41,935 (69.3%)	51,407 (63.8%)	17,363 (63.6%)	10,433 (64.8%)	14,079 (70.8%)	23,671 (66.8%)
Age, mean (SD)	48.6 (10.0)	47.1 (9.4)	47.3 (9.4)	47.7 (9.7)	51.1 (10.0)	48.1 (9.5)
Serum creatinine, mean (SD) µmol/L	0.76 (0.20)	0.75 (0.19)	0.77 (0.24)	0.78 (0.23)	0.80 (0.31)	0.77 (0.26)
estimated glomerular filtration rate, mean (SD) mL/min/1.73 m^2^	81.8 (14.8)	82.1 (14.6)	80.9 (14.6)	79.8 (14.4)	77.8 (14.6)	81.1 (14.6)
Chronic Kidney Disease Stage, *n* (%)						
Stage G1 or G2	57,543 (95.1%)	76,679 (95.2%)	25,788 (94.5%)	15,035 (93.3%)	18,167 (91.3%)	33,455 (94.5%)
Stage G3a	2,750 (4.5%)	3,580 (4.4%)	1,346 (4.9%)	993 (6.2%)	1,568 (7.9%)	1,812 (5.1%)
Stage G3b	193 (0.3%)	218 (0.3%)	118 (0.4%)	57 (0.4%)	115 (0.6%)	123 (0.3%)
Stage G4-5	49 (0.08%)	45 (0.06%)	33 (0.12%)	25 (0.16%)	39 (0.20%)	24 (0.07%)
Urinary protein, *n* (%)						
+-	7,470 (12.3%)	9,815 (12.2%)	3,489 (12.8%)	2,008 (12.5%)	2,155 (10.8%)	4,586 (13.0%)
+ or more	4,544 (7.5%)	6,677 (8.3%)	2,257 (8.3%)	1,310 (8.1%)	1,615 (8.1%)	2,399 (6.8%)
Body mass index, mean (SD) kg/m^2^	21.9 (3.2)	23.1 (3.7)	23.6 (3.8)	23.8 (3.8)	23.1 (3.4)	23.0 (3.7)
Systolic blood pressure, mean (SD) mm Hg	120 (18)	121 (18)	122 (18)	121 (17)	122 (17)	122 (17)
Diastolic blood pressure, mean (SD) mm Hg	74 (13)	75 (13)	75 (13)	75 (13)	75 (12)	76 (13)
Abdominal circumference, male ≥85, female ≥90 (%)	12,416 (20.5%)	23,959 (30.0%)	8,793 (32.2%)	5,400 (33.5%)	5,880 (29.6%)	10,354 (29.2%)
Diabetes,^a^ *n* (%)						
high, no drugs	4,930 (8.1%)	7,330 (9.1%)	2,677 (9.8%)	1,438 (8.9%)	1,989 (10.0%)	3,376 (9.5%)
normal with drugs	144 (0.24%)	273 (0.34%)	109 (0.40%)	118 (0.73%)	235 (1.2%)	159 (0.45%)
high with drugs	882 (1.5%)	1,681 (2.1%)	775 (2.8%)	621 (3.9%)	1,189 (6.0%)	940 (2.7%)
Dyslipidemia,^b^ *n* (%)						
high, no drugs	10,000 (16.5%)	16,585 (20.6%)	6,027 (22.1%)	3,275 (20.3%)	3,286 (16.5%)	6,866 (19.4%)
normal with drugs	1,742 (2.9%)	2,937 (3.6%)	1,141 (4.2%)	869 (5.4%)	1,481 (7.4%)	1,478 (4.2%)
high with drugs	701 (1.2%)	1,541 (1.9%)	620 (2.3%)	439 (2.7%)	579 (2.9%)	728 (2.1%)
High uric acid or prescribed antihyperuricemic drugs, *n* (%)	2,096 (3.5%)	3,456 (4.3%)	1,267 (4.6%)	780 (4.9%)	773 (3.9%)	1,400 (4.0%)
The improvement of healthy behaviors after 1 year^c^						
Quitting smoking	1,286 (2.1%)	1,849 (2.3%)	646 (2.4%)	370 (2.3%)	326 (1.6%)	518 (1.5%)
Undertaking physical activity	3,236 (5.3%)	4,176 (5.2%)	2,189 (8.0%)	1,930 (12.0%)	1,707 (8.6%)	291 (0.8%)
Decrease in the amount of drinking	5,818 (9.6%)	7,943 (9.9%)	2,861 (10.5%)	1,630 (10.1%)	1,940 (9.8%)	207 (0.6%)
Decrease in the frequency of drinking	3,967 (6.6%)	5,791 (7.2%)	2,110 (7.7%)	1,185 (7.4%)	1,404 (7.1%)	372 (1.1%)
Change to normal weight ​ (body mass index greater than or equal to 18.5 ​ to 24.9 kg/m^2^)	2,250 (3.7%)	3,056 (3.8%)	1,070 (3.9%)	616 (3.8%)	634 (3.2%)	1,331 (3.8%)

SD, standard deviation.

^a^Diabetes “high”: fasting blood sugar ≥110 mg/dL or hemoglobin A1c ≥5.6%.

^b^Dyslipidemia “high”: triglyceride ≥150 mg/dL or high-density lipoprotein cholesterol <40 mg/dL.

^c^Even though the questionnaire in the next year contained missing values, we did not remove it from the denominator.

**Figure 2.**
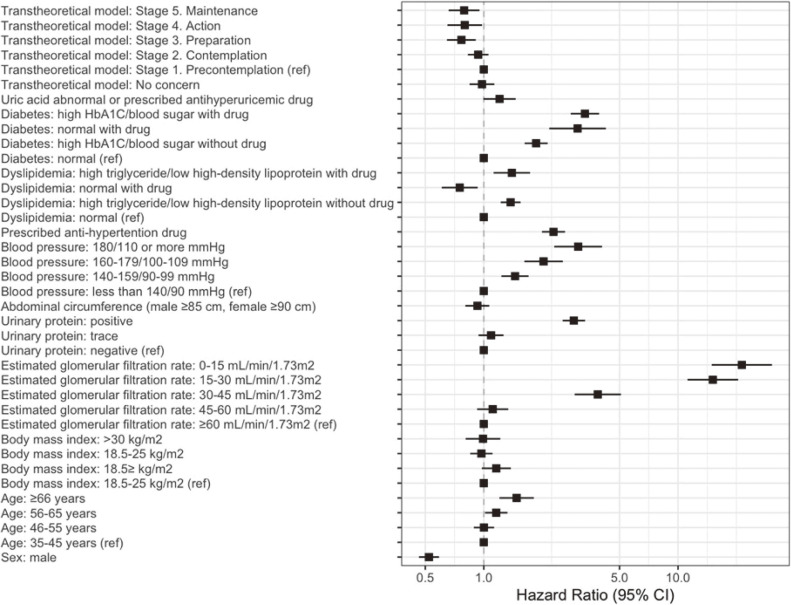
Hazard ratios for a decrease of 30% or more in estimated glomerular filtration rate: stages of transtheoretical model, sex, age, body mass index, estimated glomerular filtration rate, urinary protein, abdominal circumference, blood pressure, dyslipidemia, diabetes, and uric acid

Compared with the stage 1 group, the risk of decreasing renal function was significantly lower in the stage 3 group (HR 0.77; 95% CI, 0.65–0.91); in the stage 4 group (HR 0.80; 95% CI, 0.65–0.98); and in the stage 5 group (HR 0.79; 95% CI, 0.66–0.95), after adjusting for age, sex, eGFR, body mass index, blood pressure, blood sugar, dyslipidemia, uric acid, urinary protein (Table 2). The forests plots of the HRs of other covariates are shown in Figure 2, which shows that urinary protein, diabetes, blood pressure, age, and lower eGFR were associated with decreasing renal function.

**Table 2.** Cox proportional hazards regression models showing the effects on the risk of estimated glomerular filtration rate decline

	Number of outcomes	Incident rate per 1,000 person-years (95% CI)	Hazard ratios in model 1^a^	Hazard ratios in model 2^b^
Stage 1: Precontemplation (not ready)	461/60,535	0.73 (0.68–0.78)	reference	reference
Stage 2: Contemplation (getting ready)	608/80,522	0.66 (0.62–0.70)	0.97 (0.86–1.10)	0.94 (0.83–1.06)
Stage 3: Preparation (ready)	190/27,285	0.65 (0.62–0.68)	0.82 (0.69–0.97)	**0.77 (0.65–0.91)**
Stage 4: Action (current action)	117/16,110	0.60 (0.55–0.66)	0.83 (0.67–1.02)	**0.80 (0.65–0.98)**
Stage 5: Maintenance (monitoring)	161/19,889	0.61 (0.54–0.68)	0.87 (0.72–1.04)	**0.79 (0.66–0.95)**
Unknown: No concern	299/35,414	0.70 (0.63–0.77)	1.04 (0.90–1.20)	0.98 (0.85–1.13)

CI, confidence interval.

Model 1: adjusted for age, sex, body mass index, estimated glomerular filtration rate at baseline, and urinary protein. (without medication factors).

Model 2: adjusted for model 1 plus blood pressure, blood sugar, dyslipidemia, and uric acid. (with medication factors).

**Figure 3.**
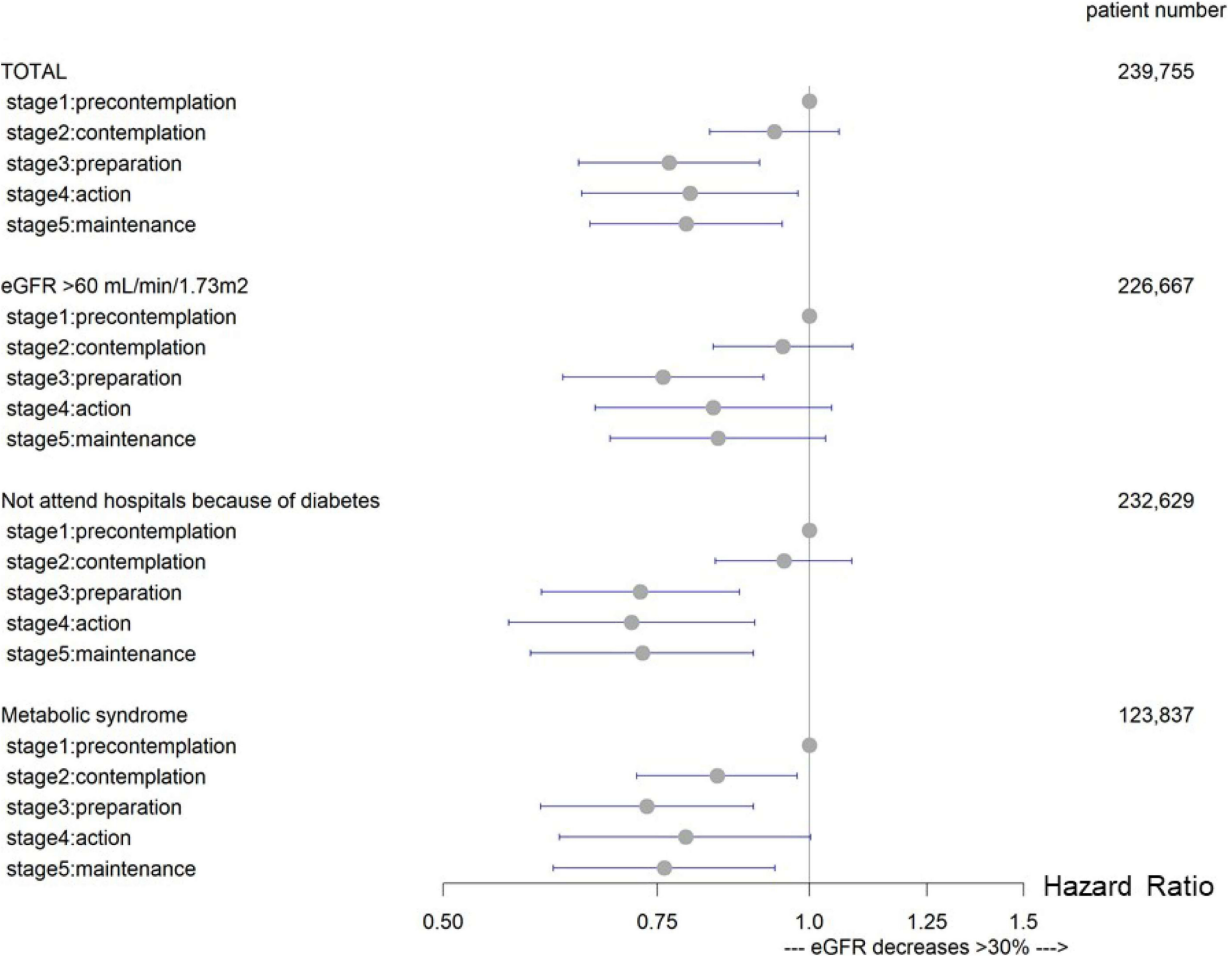
Subgroup analysis: eGFR categorized by >60 mL/min/1.73 m^2^; who did not attend hospital because of diabetes (without diabetes drug prescription); who met one or more criteria for Japanese metabolic syndrome

The major results of the subgroup analysis are shown in Figure 3. When we included 226,667 employees whose eGFR was >60 mL/min/1.73 m^2^, the hazard ratios of decreasing renal function were 0.95 (95% CI, 0.83–1.09) in the stage 2 group, 0.76 (95% CI, 0.63–0.92) in the stage 3 group, 0.83 (95% CI, 0.67–1.04) in the stage 4 group, and 0.84 (95% CI, 0.69–1.03) in the stage 5 group, compared with the stage 1 group. When we included 12,049 employees whose eGFR was 45–60 mL/min/1.73 m^2^, the hazard ratios of decreasing renal function were 0.78 (95% CI, 0.49–1.26) in the stage 2 group, 0.81 (95% CI, 0.45–1.48) in the stage 3 group, 0.19 (95% CI, 0.06–0.61) in the stage 4 group, and 0.65 (95% CI, 0.34–1.22) in the stage 5 group, compared with the stage 1 group. When we included 1,039 employees whose eGFR was ≤45 mL/min/1.73 m^2^, the hazard ratios of decreasing renal function were 0.98 (95% CI, 0.60–1.58) in the stage 2 group, 0.87 (95% CI, 0.50–1.52) in the stage 3 group, 1.19 (95% CI, 0.63–2.23) in the stage 4 group, and 0.70 (95% CI, 0.40–1.23) in the stage 5 group, compared with the stage 1 group. The trend of point estimates did not change fundamentally in other subgroups.

The sensitivity analysis also showed similar hazard ratios. When we excluded employees aged 60 or more years, the results were 0.95 (95% CI, 0.82–1.09) in the stage 2 group, 0.77 (95% CI, 0.63–0.94) in the stage 3 group, 0.83 (95% CI, 0.65–1.05) in the stage 4 group, 0.75 (95% CI, 0.60–0.95) in the stage 5 group, and 0.99 (95% CI, 0.84–1.18) for the unknown stage group, compared with the stage 1 group. When we excluded employees who had taken any medication for hypertension, diabetes, or dyslipidemia, the results were 1.07 (95% CI, 0.91–1.25) in the stage 2 group, 0.79 (95% CI, 0.63–1.00) in the stage 3 group, 0.68 (95% CI, 0.50–0.93) in the stage 4 group, 0.75 (95% CI, 0.56–0.99) in the stage 5 group, and 1.14 (95% CI, 0.94–1.37) in the unknown stage group, compared with the stage 1 group.

## DISCUSSION

We found that persons in stages 3–5 had a habit of healthier behaviors with a lower risk of eGFR decline after adjusting for confounding factors, compared with those who were in stage 1. In particular, those who were in stage 3 (preparation stage) showed less eGFR decline than those in stages 4 or 5 (action, maintenance stage), whereas those in stage 4 or 5 had a slightly higher risk of eGFR decline than those in stage 3.

TTM is a therapeutic theory that led people to realize the importance of healthier behaviors according to their consciousness of the behaviors.^4^ In several studies, TTM theory has been applied to subjects with lifestyle diseases and improved their behaviors on weight management, adherence to antihypertensive medication, and adherence to lipid-lowering drugs.^5^^–^^7^

The present study, with 1-year follow-up questionnaires, is shown in Table 1. It demonstrated that those in stages 3–5, but not in stages 1–2, improved their various kinds of behaviors. Although our study targeted the general population, similar findings were observed in CKD patients. In fact, a systematic review revealed that undertaking physical activity is correlated with mortality rates and the reduction of adverse clinical events in CKD patients,^12^ suggesting that such healthy behaviors contribute to slowing eGFR decline in stages 3–5. Currently, the KDIGO guideline recommends that CKD patients undertake more physical activities.

Whereas those in stages 3–5 were associated with a lower decline of renal function, those in stage 3 had better prognosis than stages 4–5. The difference could be explained by the status of physical activity and diet. According to Prochaska,^4^ stage 3 is defined as “the stage in which people are intending to take action in the immediate future, usually measured as the next month”. This means that employees in stage 3 do not conduct physical actions and diets but intend to improve their behavior. Therefore, improvement of their behavior would be achieved and lead to a beneficial outcome. In contrast, those in stages 4–5 are subjects who have been already made significant modifications to their lifestyle such that they have little room to further improve their behaviors.

The benefit of TTM-based intervention for improving health outcomes remains controversial. In fact, some researchers often failed to show a positive effect.^13^^,^^14^ As a result, a Cochrane systematic review could not conclude that TTM-based intervention might be effective in weight loss.^15^ This discrepancy could be accounted for in part by the distinct target stages. A TTM-based method may lead us to classify target stages of TTM in which patients’ health conditions could improve effectively in terms of the results of laboratory tests, such that we can promote patients’ awareness of healthy behaviors to elevate their TTM-stages. In our case, an analysis was performed for subjects in each stage. As a result, we found positive results only in stages 3–5, suggesting that the effect might vary between each stage. Therefore, when all stages were combined in other reports, a positive effect in any specific stage may have been canceled out by no effect in other stages. Consistently, a previous randomized trial showed that HbA1c was significantly reduced in diabetic patients in pre-action stages, whereas such an effect was canceled when subjects in all stages were analyzed together.^16^ Our results would imply that targeting specific stages would improve the results of laboratory tests effectively.

The next issue is how to move patients from stages 1–2 to stages 3–5 in clinical settings. In this regard, Prochaska et al developed a TTM-based intervention aiming to lead patients to move onto different stages.^4^ They showed that four processes are important to change stages: “Consciousness raising”, getting the facts; “Dramatic relief”, paying attention to feelings; “Environmental reevaluation”, noticing your effect on others; and “Self-liberation”, making a commitment. Perhaps, it may be of importance for us to educate patients to understand what healthy behaviors are, how to change and consolidate their behaviors, and what they can do for the health of those around them. These processes could proceed to a change of healthy behaviors to reduce kidney injury.

In addition to improving the healthy behaviors, maintaining the habit is another critical issue in traditional cognitive behavior therapies. Cooper et al examined the effect of cognitive behavioral treatment on body weight in obese people. It was found that the effect of behavioral therapy was transient, and the great majority regained almost all the weight that they had lost with behavioral treatment during the 3 years in the randomized controlled trial,^17^ indicating that maintaining healthy behaviors would be difficult. Alternatively, several researchers have indicated that the new cognitive therapies, including acceptance-based behavioral treatment or mindful intervention, might be an option to improve the maintenance of healthy behaviors.^18^^,^^19^ Future studies might discover more effective methods of intervention.

There were several limitations to our study. First, we dealt with all the competing events, including acute kidney injury (AKI), as censored events, and it might cause healthy worker bias. However, AKI might occur regardless of the stages of behavioral change, AKI incidence is reported at a low rate of 500 persons per 1,000,000,^20^ and all-cause mortality in patients with AKI is estimated as one in four or less.^21^ Second, our study did not consider several unmeasured confounding factors, including dietary or exercise habits. Further studies, including a causal mediation analysis, are needed to confirm the impact of such lifestyle habits, even though it is difficult to quantify them accurately.

### Conclusion

Compared with the precontemplation stage (stage 1), the preparation, action, and maintenance stages (stages 3, 4, and 5), were associated with healthier behavior and a lower risk of eGFR decline after adjusting for confounding factors. The effect of TTM-based therapy may be clarified further in a specific population that performs healthy behaviors.
